# The Effects of Adding Different HALS on the Curing Process, Film Properties and Lightfastness of Refined Oriental Lacquer

**DOI:** 10.3390/polym12040990

**Published:** 2020-04-24

**Authors:** Chia-Wei Chang, Jia-Jhen Lee, Kun-Tsung Lu

**Affiliations:** Department of Forestry, National Chung Hsing University, 250, Kuo-Kuang Rd., Taichung 402, Taiwan; dimmerc@hotmail.com (C.-W.C.); jessie135kimo@livemail.tw (J.-J.L.)

**Keywords:** oriental lacquer, hindered amine light stabilizers, laccase-catalyzed reaction, oxidative polymerization

## Abstract

Oriental lacquer is a natural polymeric coating with a satiny texture and excellent characteristics, such as chemical resistance and durability. However, the poor lightfastness resulted in the natural aromatic structures of the urushiol structures limited its suitability for outdoor application. This study aimed at the improvement of the lightfastness by adding the different hindered amine light stafbilizers (HALS) with 2 phr addition as well as the effects on the coating and film property of the refined oriental lacquers (RL). The *Cryptomeria japonica* plate, glass sheets, and the other substrates were used for finishing. The results showed that the lightfastness of RL film was obviously improved by adding 2 phr HALS of Bis(2,2,6,6-tetramethyl-4-piperidinyl) sebacate (H90) containing -NH group and Bis (1,2,2,6,6-pentamethyl-4-piperidinyl)-[[3,5-bis(1,1-dimethyl ethyl) -4-hydroxyphenyl] methyl] butylmalonate (H60) containing -NCH_3_ groups. The HALS additions increased the pH value of RL and improved the activities of laccase-catalyzed reaction. Meanwhile, the oxidative polymerization of the side chains of RL was inhibited, caused by a radical scavenging ability of HALS. The changes in the drying process affected not only the curing time, but also the film properties. Among the 2 phr additions of different HALS, the film containing H90 had the best lightfastness. Meanwhile, it kept the most similar properties with RL and shortened the drying time of RL, and it was selected as the best HALS addition under 2 phr in this study.

## 1. Introduction

With the consumption of petrochemical raw materials, the use of natural and renewable resources as raw materials combines the principles of environmental protection and sustainable development, leads to cost reductions, and promotes the creation of a comfortable, safe, and healthy environment for humans. Oriental lacquer (OL), which is a natural polymeric material from *Rhus* trees, is regarded as a renewable resource because it can be cultivated [1]. The compositions of oriental lacquer (OL) are 60–65% of urushiol, 20–25% of moisture, 5–7% of plant gum, 2–5% of nitrogenous compounds, and approximately 1% of laccase [2]. 

The main component in OL, urushiol, is a catechol derivative with a long unsaturated hydrocarbon side chain, and the chemical structure imparts various properties to the coatings [1,3,4,5,6]. The laccase acts as a catalyst and promotes the oxidative polymerization of the urushiols [7,8]. The oxidative reaction of the catechol moiety of urushiol to form oligomers is the first step of OL curing. Meanwhile, the urushiols transform into less-antioxidative quinones through the laccase-catalyzed polymerization [9]. Finally, the unsaturated double bonds, the side chains of urushiols, undergo autoxidative polymerization to form a highly cross-linked network film. Many different refined processes were used to promote the drying process of OL because improving the laccase-catalyzed polymerization takes a long drying time in the limited condition of humidity and temperature [3,10,11,12]. These improved OL had distinct film properties and were widely applied on the arts and crafts, and generally named as refined oriental lacquer (RL). 

However, the urushiol contains 1,2,3-tri-o- or 1,3-di-o-substituted catechol and isomers containing double bonds or small amounts of saturated alkyl groups, and it possesses poor lightfastness [9]. The mechanism and appearance changes of photo-degradation of oriental lacquer had been identified by Hong et al. [13]. They explained that the degradation initiates with the oxidation of the side chains of catechols and leads to the generation of ketone groups and acids under the UV exposure. Besides, Nakagoshi and Yoshizumi [14] reported that the OL responds differently to light of various wavelengths. Coueignoux and Rivers [15] and Ogawa et al. [16] also explored the formation of microcracks of OL under the light and humidity. In the document of Honda et al. [2], they found that the chalking and cracking lead to the white spot generation on the film surface and confirmed that the emerging of plant gum causes this phenomenon. Obataya et al. [17] also discussed the effects of aging and moisture on the dynamic viscoelastic properties of oriental lacquer films. 

Many light stabilizers have been used to enhance the lightfastness of materials, such as hindered amine light stabilizers (HALS), UV absorbers, and UV-blocking particles. While the HALS had abilities to trap free radicals, deactivating peroxides, and singlet oxygen [18]. Among these light stabilizers, HALS, a series of derivative of 2,2,6,6-tetramethylpiperidine, is the most commonly used in the coating industry and was well documented, especially in the polyolefins [19]. The mechanism of the HALS stabilization is commonly accepted and it is known as the Denisov cycle [20]. The HALS initially converts to the corresponding nitroxide (N-O•), which reacts with free radicals and forms alkoxyamines (R-O-N-). Simultaneously, the HALS regenerates from the alkoxyamines through participation with peroxy radicals. Hong et al. [13] introduced the 2% HALS and UV absorber in the RL for providing the better lightfastness of film for outdoor application. While there are few studies regarding the addition of HALS decreased the cross-linking density of films and reduced the film mechanical properties. Moreover, the alkalinity of HALS raised the pH value of RL and affected the drying of laccase-catalyzed reaction and the followed radical addition. 

In our previous study [21], the effects of 1–5 phr HALS [Bis (2,2,6,6-tetramethyl-4-piperidinyl) sebacate] additions on the lightfastness improvement of RL were discussed. Notably, the HALS addition of more than 3 phr inhibited the drying of RL. While the film with 2 phr HALS addition substantially improved the lightfastness, as well as possessed superior smoothness, hardness, impact, and bending resistance of films and the other film properties were, to a certain extent, similar to those of OL. Notably, we found the RL drying seems to interact by the alkalinity of HALS. For clarifying the interaction of the alkaline of HALS, RL drying, and film properties, in the continued investigation, the RL was added with four additives of commercial HALS with different alkalic groups for improving the film lightfastness. The influences of the different HALS additives at 2 phr addition to the coating and film properties were examined, and the best formulas for RL were selected.

## 2. Materials and Methods

### 2.1. Preparation of RL

The OL was harvested from the cultivar *Rhus succedanea* and was purchased from the Long-Nan Museum of Natural Lacquer Ware, Nantou, Taiwan. The properties, including color, composition, viscosity, and molecular weight, have been analyzed in our laboratory and published [21]. The RL was made from the 400 g OL by stirring at 60 rpm, 40 °C in a glass plate tray until its water content was reduced to 3.5%. 

### 2.2. Materials

**Scheme 1:**
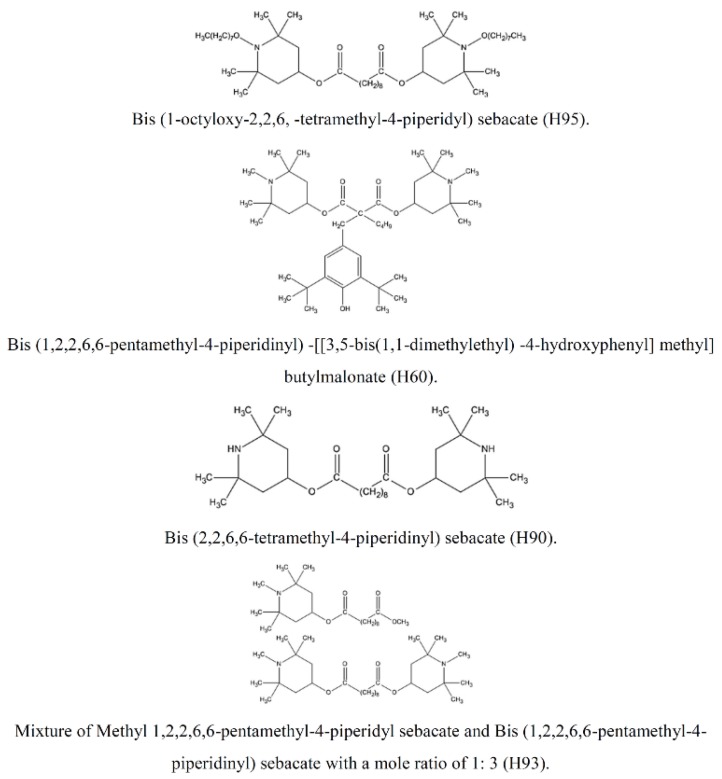
The structures of four additives of hindered amine light stabilizers (HALS).

All of the HALS were provided from Everlight Chemical Co., Taoyuan, Taiwan, as following. Bis (1-octyloxy-2,2,6-tetramethyl-4-piperidyl) sebacate with molecular weight (*M*_w_) of 737 g/mol and pH value of 5.3 was coded as H95. Bis (1,2,2,6,6-pentamethyl-4-piperidinyl) -[[3,5-bis(1,1-dimethylethyl)-4-hydroxyphenyl] methyl] butylmalonate with *M*_w_ of 685 g/mol and pH value of 7.1 was coded as H60. Bis (2,2,6,6-tetramethyl-4-piperidinyl) sebacate with *M*_w_ of 480 g/mol and a pH value of 9.1 was coded as H90. The mixture of methyl 1,2,2,6,6-pentamethyl-4-piperidyl sebacate and bis (1,2,2,6,6-pentamethyl-4-piperidinyl) sebacate with a mole ratio of 1:3 had an averaged *M*_w_ of 404 g/mol, respectively, and a high pH value of 9.1 was named as H93. Scheme 1 shows the structures of all HALS.

### 2.3. Substrates for Finishing

*Cryptomeria japonica* is a common plantation species and it represents approximately 10% of the total plantation area in the mountain areas of Taiwan. Therefore, *Cryptomeria japonica* boards (with a moisture content of 11.0%, 15 cm × 10 cm × 1 cm, radial section) was used as the finishing substrates. Glass sheets (15 cm × 10 cm × 0.2 cm), S-16 wear-resistant steel plates, tin-coated iron sheets (20 cm × 5 cm × 0.1 cm), and Teflon sheets (15 cm × 15 cm × 0.1 cm) were used as the experimental substrates. All of the substrates were prepared, as specified by the CNS 9007 Standard.

### 2.4. The formulation of HALS-Containing RL

Different HALS were added into the RL with 2 phr, according to the solid-content of RL. The HALS was dissolved in 10 mL xylene with supersonic oscillation, and then the HALS solution was added to the RL and stirred evenly for 10 min. The codes of RL that contained different HALS were coded by RL-HALS; for instance, the RL contained 2 phr H90 was coded as RL-H90.

### 2.5. The Measurement of Coating Properties

The pH value was measured at 25 °C while using a Suntex sp-701 probe (Suntex Instruments, Taipei, Taiwan). The Viscosity was determined at 25 °C by a Brookfield DV-E Viscometer (Brookfield Engineering Laboratories, Middleboro, MA, USA). The drying time was measured by a three-speed BK Drying Time Recorder (BYK Additives & Instruments, Wesel, Germany). The long glass sheets were coated by RL with a wet film thickness of 76 µm. The measurement proceeded at a constant temperature of 25 °C and a constant humidity of 80% RH. The drying stats was distinguished into a dust-free dry (DF), touch-free dry (TF), and hardened dry (HD) [22].

### 2.6. The Measurement of Film Properties 

The substrates were coated with a wet film of 100 μm thick by a universal applicator (B-3530, Elcometer, Manchester, UK). All of the complete specimens were cured at a constant temperature of 25 °C and humidity chamber of 80% relative humidity for one day, and the cured films were subsequently placed at an air-conditioned environment at 26 °C and 65 ± 5% RH for another one week. The film properties were tested after this drying process. The film lightfastness of wood specimen was carried out with a Paint Coating Fade Meter (Suga Test Instruments Co., Tokyo, Japan) at a chamber temperature of 32 ± 4 °C, and the light source was mercury light (H400-F). After 0, 12, 24, 48, 96, 144, and 192 h exposure, the color changes of nine points of each specimen were measured by a spectrophotometer (CM-3600d, Minolta. Osaka, Japan) that was fitted with a D_65_ light source with a test-angle of 10° and an 8 mm test-window. The CIE L*, a*, and b* color parameters were computed, the color difference (ΔE*), and the brightness difference (ΔL*), and yellowness difference (ΔYI) were obtained from the Minolta MCS software system. The scanning electron microscope (SEM) analysis was performed while using an electron microscope (SM-200, Topcon, Tokyo, Japan) with a magnification of 1350×. The Fourier transform infrared spectroscopy (FTIR) measurements of films were performed using a Perkin-Elmer Spectrum 100 (Perkin Elmer, Shelton, CT, USA) instrument. The coated glass specimens were measured by single-spot total reflection with a range of 4000 to 650 cm^−1^ at a resolution of 4 cm^−1^, averaged over 16 scans per sample. The gloss finished on wood and parallel to wood grain was determined by a Dr. Lange Reflectometer 60° gloss meter (REFO 3, Dusseldorf, Germany) with a repetition of 15 times. Atomic force microscope (AFM) analysis was measured by Bruker Dimension Icon (Bruker, Palaiseau, France) system with a peak force tapping mode. The experimental scope was 30 μm × 30 μm, and the average center line roughness (Ra) was obtained. The film adhesion was determined by the cross-cut method with a repeated number of 3 and, according to CNS K 6800, and the best grade is 10, followed by 8, 6, 4, 2, and 0 grade. The hardness was measured according to the DIN 53157 König standard by a König/Persoz pendulum-type hardness tester that was produced by Brave Co., UK with a repeated number of 7. Impact resistance: The impact resistance of the films was determined by depending on the passable striking height of finished wood specimen using a Dupont Impact Tester IM-601 (Du Pont Co., Wilmington, DE, USA). The falling weight was 300 g and the impact hammer diameter was 1/2 inch. For conducting the mass retention of films, approximately 0.3 g of the film was separated from the substrates and placed in a Soxhlet extractor with 250 mL acetone with a repeated number of 5. The acetone was siphoned four times per hour for 6 h continuously under heating, and then the films were taken out and dried to calculate the mass retention. The dynamic mechanical analysis was carried out by a Perkin-Elmer DMA 8000 (Perkin-Elmer, MA, USA) at 1 Hz under the tension mode in order to measure the glass transition temperature (T_g_) of films. This test was taken in a nitrogen atmosphere from 0–180 °C with a heating rate of 5 °C/min. The film tensile strengths and elongation at break values were performed by a Shimadzu EZ Test Series Tensile Tester (Shimadzu UEH-10, SHIMADZU Co., Kyoto, Japan) with a repeated number of 5. The isolated films were shaped according to ASTM D638. The experiment was carried out with an elongation speed of 5 mm/min. with a fixture distance of 40 mm. The abrasion resistance was performed on a Taber Abrasion Tester (Model 503, Taber^®^) with a CS-10 abrading wheel and 500 g loading. The averaged film mass loss of five repeated exams after 1000 turn was recorded.

## 3. Results and Discussion

### 3.1. Coating Properties of HALS-Containing RL

The films of RL contained 2 phr HALS (H90) had the best balance between the lightfastness and drying time within the additions of 1, 2, 3, 4, 5 phr, according to our previous report [21]. In the following study, different HALS, including H90, H60, H93, H95 with 2 phr were mixed with RL, and Table 1 lists their coating properties. The results showed the RL had the lowest pH value of 3.3, while the RL contained HALS were 3.6–4.8. The addition of HALS lowered the pH value of RL due to the alkaline nature of the amine groups. Because the =N-H groups were more alkaline than the =N-CH_3_ and =N-alkane [23,24], the RL-H90 had the highest pH value, while the RL-H95 was the lowest one. The HALS-containing RL had a higher viscosity than RL. The increases of viscosity had no distinct tendency with HALS molecular weights, but had a correlation with the hydrogen bonds of HALS. The secondary amines of H90 provided more hydrogen bonds than the other HALS and had the highest viscosity of 221 cps. Nevertheless, the hydrogen bonds from the hydroxyl groups and composition of the trigeminal structure increased the internal stress during flowing, even though the H60 lacked the secondary amine structure. As a result, the RL-H60 had a similar viscosity to the RL-H90 of 214 cps. The RL-H95 that had the lowest viscosity favored its linear structure and has fewer carboxyl groups than the RL-H93. In conclusion, the RL with the addition of 2 phr HALS affected the finish processes despite the slight increases of viscosity.

The drying processes of OL could be derived into two steps, including a laccase-catalyzed reaction between the phenol groups of urushiols, and following by the oxidative addition of the long-chain alkenes [2,25,26]. In the drying test, the end of two drying steps could correspond to the TF and HD, respectively, according to the previous study [22]. It could be speculated that the pH value had less effect to the RL than OL because the partial laccase-catalyzed reaction was carried out in the refined process [27,28]. Even though the drying test showed that the HALS-containing RL with a pH value of 4.6 had the shortest TF, the results were similar to the report of Hong et al [13], and advanced that the RL had the shortest drying time with pH values around 4–5. We found the HALS-containing RL had HD around 4–5.5 h, which were lower than the 6.0 of RL. The results showed that the addition of 2 phr HALS resulted in a shorter drying time and seemed to have no apparent adverse effects on the oxidative addition of RL. However, there had been a reverse result when the RL contained 3 phr or higher of HALS in our previous study [21]. Interestingly, the adding of H95 shortens 0.5 h of TF when compared to the RL, but it didn’t further improve the following curing process. The time of RL-H95 reaching to HD from the TF was 2.5 h, and the RL also took 2.5 h from the TF to HD. The result suggested that the oxidative addition of RL might be slowed by the addition of H95.

### 3.2. Lightfastness of HALS-Containing RL Films

**Figure 1:**
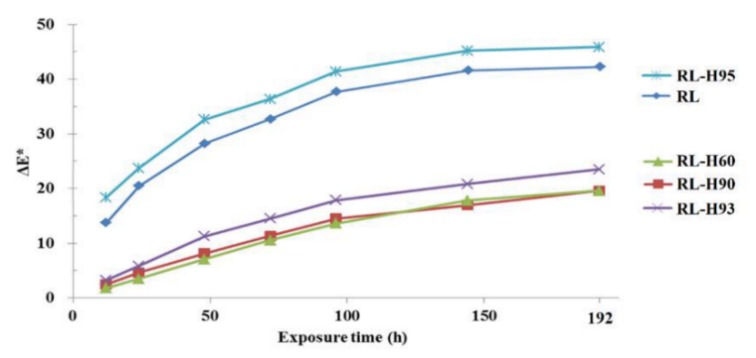
Time dependent color difference (ΔE*) of RL films with different HALS additives.

Figure 1 draws the time-depending color difference (ΔE*) of RL films that contained different HLAS in the 192 h lightfastness test, and Table 2 lists the color-changed parameters. The results showed that the values of ΔE* were increased with the exposure time. The RL had a ΔE* of 42.3, and the RL-H95 had a similar ΔE* of 45.9. A few amounts of differences between their ΔE* values indicated that the adding of H95 did not improve the lightfastness of RL films. Meanwhile, the films that contained H60, H93, and H90 had lower ΔE* values of 19.6–23.5 than RL after 192 h exposure time. When the HLAS was applied in the acidic condition, the reduction or even deactivation was confirmed [24]. However, during the RL drying from the liquid to the solid films, the urushiol additive underwent a laccase catalyzed reaction that lowered the amounts of phenol groups [22], and the acidic nature of RL was gradually decreased. It suggested the improvement of the lightfastness of RL-60, RL-90, RL-93, even though their coatings were acidic. However, this was still unable to explain the poor lightfastness of RL-95. Among all HALS, H95 had a long alkane, which caused the poor transferability [29]. Besides, the =N-alkane groups of H95 had lower reactive activity than the =N-H or =N-CH_3_ groups, and low reactive group concentration (the *M*_w_ of H95 is 366 g). The H60 had similar reactive group concentration (the *M*_w_ of H60 is 342 g). However, its reactive groups, =N-H, had more reaction activity than the =N-alkane groups of H95. Consequently, the addition of 2 phr H95 did not enhance the lightfastness of RL films, while the RL-H60 showed an excellent film lightfastness. Interestingly, between the N-CH_3_ groups-containing HALS of H93 and H60, the H93 had higher reactive group concentration, but the RL-H93 showed lower lightfastness than RL-H90. In this examination, this phenomenon suggested the HALS effect was determined by the transferability, rather than the reactive group concentration. 

**Figure 2:**
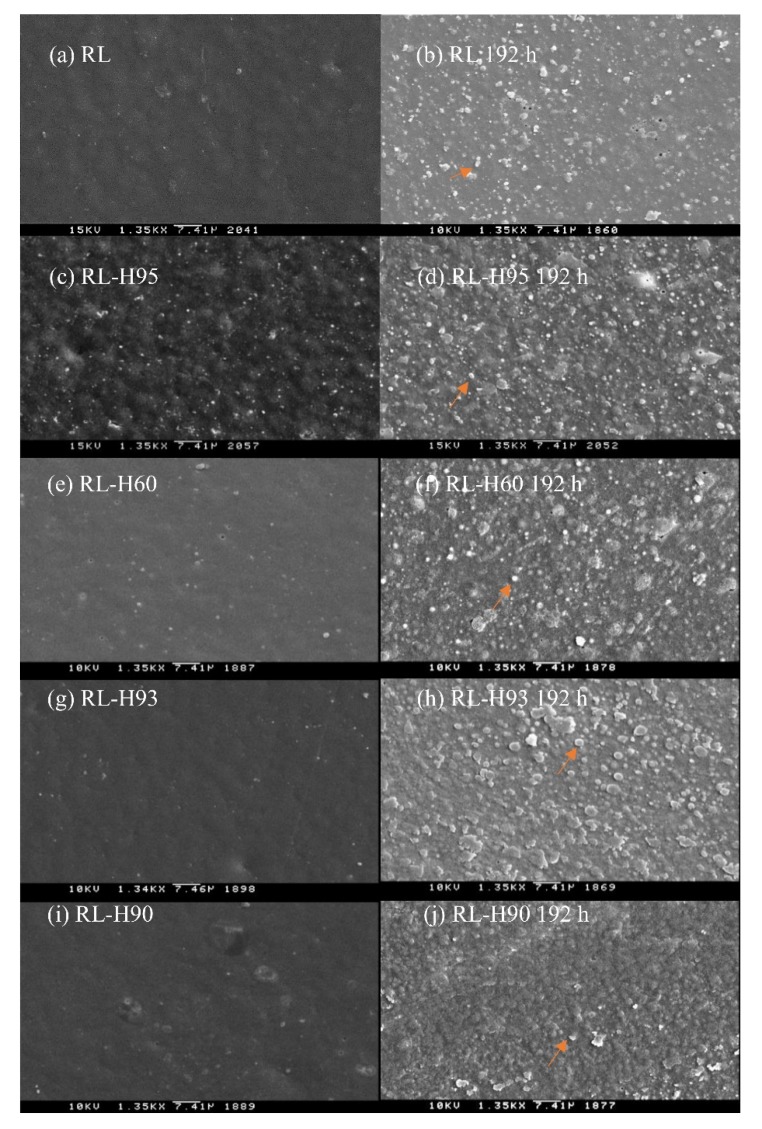
Scanning electron microscopic image (1350x) of RL films with different HALS additives after UV exposure test. (**a**) RL (**b**) RL after 192 h exposure (**c**) RL-H95 (**d**) RL-H95 after 192 h exposure (**e**) RL-H60 (**f**) RL-H60 after 192 h exposure (**g**) RL-h93 (**h**) rl-h93 after 192 h exposure (**i**) RL-H90 (**j**) RL-H90 after 192 h exposure.

The increasing brightness difference (ΔL*) of all the specimens exhibited the appearances of films were whiter after 192 h exposure. The appearance changes were most commonly observed in OL and RL and they were associated with the migration of plant gum to the film surface [30]. In the SEM analysis, as shown in Figure 2, more particles formed by plant gum were observed in the RL-H95 than RL after the 192 exposure. The plant gum moved easier to the surface from a low cross-linking structure assembled film. In summary of the curing time, the SEM and brightness ΔL* results suggested that the addition of H95 had a negative effect on the film structure. 

### 3.3. FTIR Analysis of HALS-Containing RL Films

**Figure 3:**
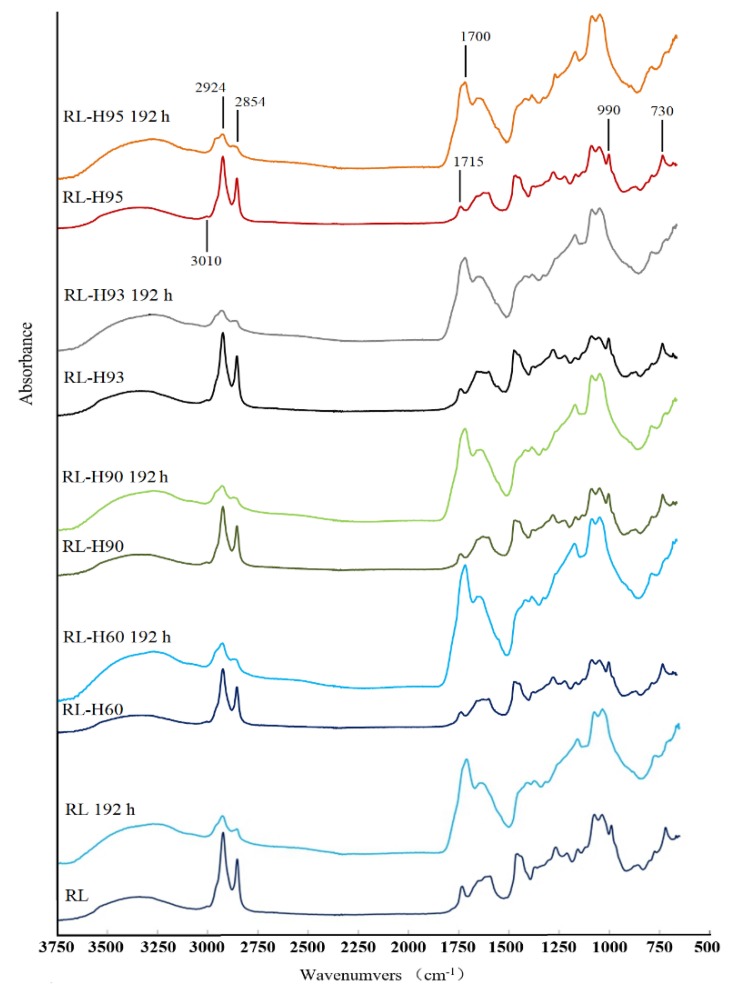
Fourier transform infrared spectroscopy (FTIR) spectra of RL films with different HALS additives.

The FTIR illustrated in Figure 3 was used to monitor the changes of functional groups of different HALS-containing RL. The spectra of HALS-containing RL were similar to that of the RL, the main functional groups, including -OH_(v)_ (3200–3400 cm^−1^), alkene-H_(δ)_ (3010 cm^−1^), CH_2(v)_ (asymmetric, 2924 cm^−1^; symmetric 2854 cm^−1^), alkene-H_(v)_ of benzene ring (1624, 1595, 730 cm^−1^), -C-O-_(v)_ (1715 cm^−1^), -C=O_(v)_ (1700 cm^−1^), and conjugated triene of RL side chains (990 cm^−1^). These groups were confirmed with the previous study [22,26,31]. While the functional groups of HALS were not seen in the spectra because they were low concentration (2 phr) in films and overlapped with the functional groups of RL. After the lightfastness experiment, the functional groups, including Alkene-H_(δ)_, CH_2(v)_, were decreased by photo-degradation. Besides, the characteristic peaks of benzene rings were lowered due to the quinones that were produced from the photochemical reaction. Besides, the -C-O-_(v)_ (1700 cm^−1^) peak that was associated with the additional derivatives of urushiols and cross-linking bonds from the oxidative addition was weakened. Simultaneously, a new peak at 1715 cm^−1^ related to the -C=O_(v) of_ carbohydrate appeared. These results explained the degradation and damage of films, and then the plant gum migrated to the surface of the film. Previous studies confirmed the phenomenon [30,31]. 

The peak at 990 cm^−1^ was lowered after the lightfastness experiment and indicated that the uncured conjugated triene was consumed during the UV exposure. We inferred this consumption was due to the post oxidative addition; however, the FTIR results showed that it was unable to avoid the degradation of the film in the 192 h lightfastness experiment as well as the long-term outdoor exposure. In conclusion, the CH_2(v)_ peak at 2924 cm^−1^ might be associated with the degradative degree of films, and the results showed the RL-H60 had kept most peak strength and indicated that the RL-H60 had the best lightfastness, while the RL-H95 was the worst.

### 3.4. Film Properties of HALS-Containing RL

Table 3 and Table 4 list the film’s properties. We found that the gloss of films had a visible change when the RL was added with different HALS. The HALS-containing RL had a higher gloss of films in addition to the RL-H95. For clarifying the gloss changes that were associated with the flowing properties or the stack of different polymer structures in the curing process, we used the AFM to analyze the μm-scaled appearances of films. We found the R_a_ values that were obtained by the AFM had a high correlation with its film gloss. Usually, the films that are cured through the oxidative addition possess a disparity between the surface and the interior, and this disparity is more evident in the thick films due to the oxygen gradient caused by penetration in the films [32]. Consequently, a film with a strong internal force and wrinkled appearance was obtained. 

In our study, the HALS additives raised the pH value of RL and accelerated the laccase-catalyzed reaction. In contrast, the HALS additives also inhibited the oxidative addition. Therefore, the films of HALS-containing RL had a lower difference between the inner and surface and a lower R_a_ value than the RL. On the other hand, the RL-H95 had a similar pH value and drying time to the RL, so they had nearly the same R_a_ value of 95.3 nm and 88.5 nm. The RL contained HALS had a better film adhesion of eight degrees than the RL. This improvement was attributed to the decrease of internal stress and the low variation between the surface and inner of film. 

**Figure 4:**
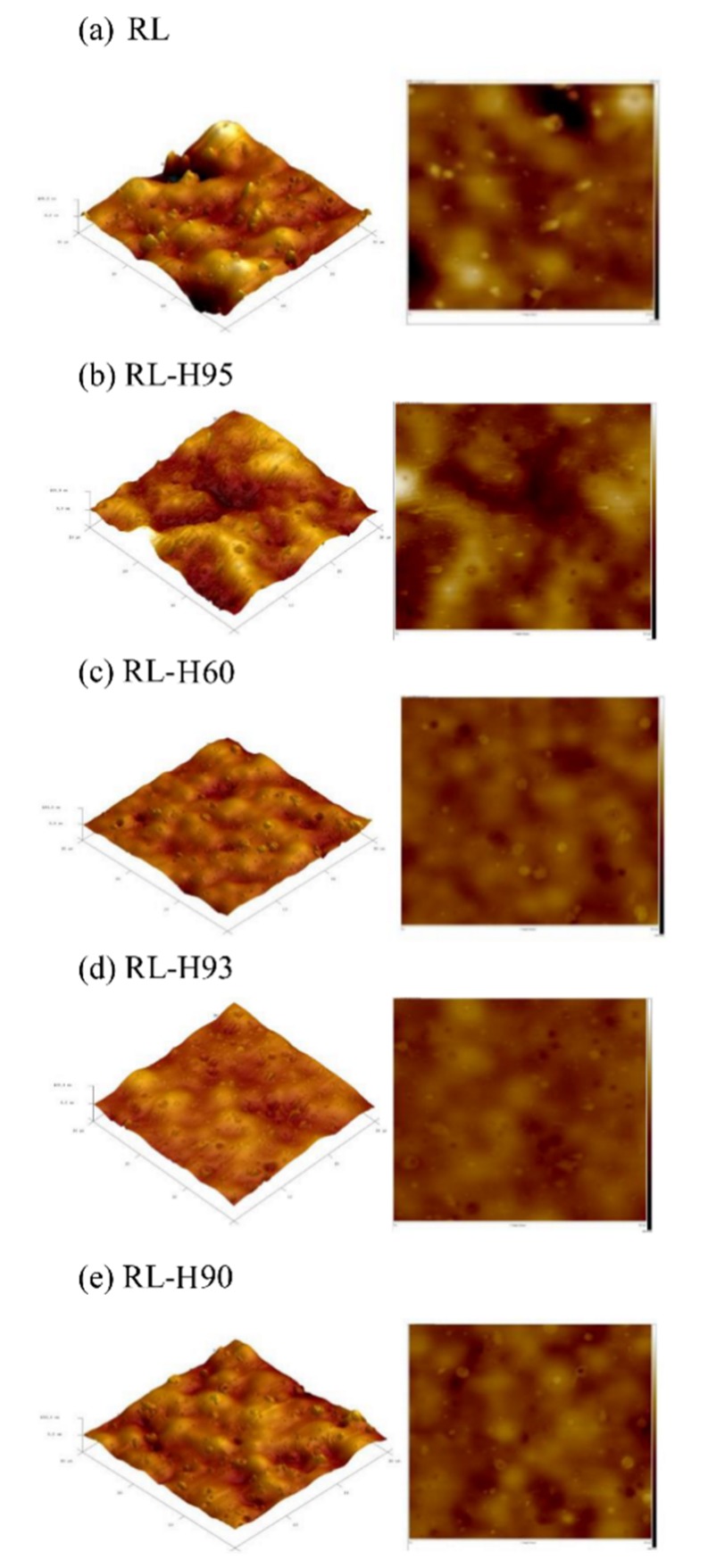
Atomic force microscope (AFM) analysis of RL films with different HALS additives (**a**) RL (**b**) RL-H95 (**c**) RL-H60 (**d**) RL-93 (**e**) RL-H90.

The HALS-containing RL had a slightly higher film hardness of 111–116 s and a slightly lower impact resistance of 10 than the values of 107 s and 5 of RL in Figure 4. The mass retention and T_g_ were used to examine the cross-linking density of films and showed uniformity results that the films of HALS-containing RL exhibited lower mass retention and T_g_, and confirmed that the addition of HALS inhibited the oxidative addition of the side chains of RL. Additionally, the alkalinity of HALS improved the laccase-catalyzed reaction and resulted in greater phenoxybenzoic linkages. Therefore, the films containing the HALS additives had a lower tensile strength, elongation at break, and abrasion resistance than the RL. Overall, the RL-H90 kept a similar hardness, tensile strength, and abrasion resistance to RL, and had the highest lightfastness, gloss, and elongation at break among the HALS-containing RL, and it was selected as the best HALS under the 2 phr addition.

## 4. Conclusions

The lightfastness of RL with adding different HALS was discussed. The examinations of curing time and film properties showed that the alkalinity of HALS improved the laccase-catalyzed reaction, whereas it inhibited the oxidative addition during the RL drying. It had negative influences on the film properties, including impact resistance, mass retention, T_g_, elongation at break, and abrasion resistance; however, the gloss, adhesion, and hardness were enhanced by additions of HALS. We observed that the photo-degradation behavior of HALS-containing RL was similar to the RL. The films were damaged first by exposure, and then the plant gum moved to the surface of films. However, the HALS-containing films had better resistance against UV exposure and less migration of plant gum than the RL films, except for the addition of H95. The addition of H90 raised the lightfastness of films. Meanwhile, it kept the most similar properties with RL and it was selected as the best HALS under the 2 phr addition.

## Figures and Tables

**Table 1:** Properties and curing parameters of refined oriental lacquers (RL) with different HALS additives.

HALS Type	pH	Viscosity (cps, 25 °C)	Curing Time (h, 25 °C, 80%RH)	
			TF ^a^	HD ^b^
RL	3.3	121	3.5	6.0
RL-H95	3.6	155	3.0	5.5
RL-H60	4.6	214	2.0	4.0
RL-H93	4.6	198	2.0	4.0
RL-H90	4.8	221	2.5	4.0

^a^ TF: Touch-free dry. ^b^ HD: Hardened dry.

**Table 2:** Lightfastness of RL films with different HALS additives after 192 h UV exposure test.

HALS Type	After 192 h UV Exposure		
	ΔE*	ΔL*	ΔYI
RL	42.3	17.9	102.8
RL-H95	45.9	21.3	79.0
RL-H60	19.6	4.6	78.8
RL-H93	23.5	6.1	90.0
RL-H90	19.6	4.6	78.5

**Table 3:** Film properties of RL films with different HALS additives.

HALS Type	60°Gloss (%)	Ra (nm)	Adhesion (grade)	Hardness (könig, s)	Impact Resistance (300 g, cm)
RL	48 ± 1	88.5	10	107 ± 3	5
RL-H95	37 ± 1	95.3	8	111 ± 1	10
RL-H60	58 ± 1	41.8	8	116 ± 1	10
RL-H90	55 ± 1	53.7	8	112 ± 1	10
RL-H93	68 ± 2	39.6	8	113 ± 1	10

**Table 4:** Film properties of RL films with different HALS additives.

HALS Type	Mass Retention (wt %)	Tg (°C)	Tensile Strength (MPa)	Elongation at Break (%)	Abrasion Resistance (mg/1000 circles)
RL	91.4 ± 0.5	99	18.8 ± 1.0	15.6 ± 0.6	12.7 ± 1.6
RL-H95	89.4 ± 0.2	91	15.1 ± 2.2	6.3 ± 1.3	13.3 ± 2.1
RL-H60	90.4 ± 0.4	97	10.3 ± 1.2	3.8 ± 0.9	36.7 ± 0.7
RL-H90	90.3 ± 1.1	94	18.8 ± 2.7	7.7 ± 1.5	15.9 ± 1.4
RL-H93	89.7 ± 0.2	91	14.6 ± 0.6	5.8 ± 0.7	24.4 ± 3.1
